# Predictive model for severe intraventricular hemorrhage risk in preterm infants: a systematic review and meta-analysis

**DOI:** 10.3389/fped.2026.1785442

**Published:** 2026-04-07

**Authors:** Zhiheng Zhan, Jing Zhang, Hui Rong, Fei Shen, Qingqing Chong

**Affiliations:** 1 Department of Respiratory, Children’s Hospital of Nanjing Medical University, Nanjing, China; 2 Department of Neonatology, Children’s Hospital of Nanjing Medical University, Nanjing, China; 3 Department of Orthopaedics Surgery, Children’s Hospital of Nanjing Medical University, Nanjing, China

**Keywords:** intraventricular hemorrhage, meta-analysis, prediction model, preterm infants, PROBAST, systematic review

## Abstract

**Objective:**

Risk prediction models offer a potential approach for early identification of severe intraventricular hemorrhage (SIVH) in preterm infants, yet their clinical applicability and methodological quality remain uncertain. This systematic review aimed to identify existing SIVH prediction models in preterm infants, evaluate their performance, and assess their risk of bias and clinical applicability.

**Methods:**

We systematically searched PubMed, Web of Science, Embase, CINAHL, MEDLINE, SinoMed, CNKI, and Wan-Fang databases for relevant studies up to September 30, 2025. Data extraction followed the CHARMS framework, while risk of bias and applicability were assessed using PROBAST. Meta-regression explored heterogeneity sources. The review is registered with PROSPERO (CRD42023486813).

**Results:**

From 13,311 initially retrieved studies, 16 prediction models were included. A meta-analysis of 7 models yielded a pooled AUC of 0.805 (95% CI: 0.756–0.853). However, all studies exhibited high risk of bias, primarily in the analysis domain, with frequent shortcomings in handling of missing data (93.75%), use of univariable analysis for predictor selection (62.50%), inadequate calibration assessment (68.75%), and non-robust internal validation (50.00%). Methodologically rigorous models demonstrated better performance.

**Conclusion:**

Current SIVH prediction models show promise but require methodological improvements. Future efforts should prioritize prospective designs, optimized predictor selection, enhanced external validation, and better calibration to improve clinical utility.

**Systematic Review Registration:**

https://www.crd.york.ac.uk/PROSPERO/view/CRD42023486813, PROSPERO CRD42023486813.

## 1 Introduction

Preterm birth complications remain a leading cause of mortality in children under five years of age, prompting the World Health Organization to actively implement measures to mitigate associated health issues and mortality risks. Intraventricular hemorrhage (IVH) is one of the most severe complications in preterm infants. According to the Papile grading system (1), IVH is categorized into mild (grades I and II) and severe IVH (SIVH, grades III and IV). While mild IVH is generally associated with favorable outcomes, SIVH carries a mortality rate as high as 18% to 40% (2), and among survivors, the probability of developing cerebral palsy ranges from 19% to 50% (3, 4), posing a significant threat. Despite advances in perinatal medicine, the incidence of SIVH remains high globally. A multinational investigation across 8 high-income regions reported that the incidence of SIVH in very preterm infants ranges from 4.2% to 11.9% (5). Currently, there are no specific curative treatments available once IVH occurs, making early identification and the rigorous implementation of preventive measures the cornerstone strategy for reducing its incidence.

It is reported that approximately 50% of IVH cases occur within the first day of life in preterm infants, and about 90% occur within the first 3 days. In 20%–40% of cases, the hemorrhage may progress during the first postnatal week, indicating that the critical window for IVH management falls primarily within the perinatal period (6). Most cases of IVH in preterm infants are clinically asymptomatic and can only be detected through imaging. Evidence from the Canadian Paediatric Society recommends an initial head ultrasound (HUS) for all preterm infants born at a gestational age of <32 weeks between 4 and 7 days after birth (7). In contrast, the American Academy of Pediatrics recommends routine cranial ultrasound around 7 to 10 days of life for infants born at ≤30 weeks, reserving screening within the first 7 days only for preterm infants with signs suggestive of significant brain injury (8). Clinical practice in China confirms that the timing of existing HUS screening may be delayed relative to the typical onset of IVH (9). Furthermore, while HUS is generally considered non-invasive, some literature suggests that premature and frequent cranial ultrasound monitoring might increase the risk of IVH, particularly for periviable extremely preterm infants (10). Therefore, developing methods to predict major hemorrhage before it occurs is of paramount importance, as it would enable healthcare providers to implement timely, targeted interventions.

Risk prediction models, which estimate the probability of a specific disease or outcome by incorporating multiple predictors, hold significant value in disease identification and prevention. Accurate risk prediction can also inform clinical resource planning and cost-effectiveness management. The predictive performance of the growing number of risk prediction models for SIVH in preterm infants remains unestablished, and no model is currently recommended for clinical practice. Therefore, a systematic assessment of their quality and applicability is warranted. This study aims to systematically review published risk prediction models for SIVH in preterm infants to provide valuable insights for clinical practice and future research.

## 2 Methods

All stages of this study were conducted in strict adherence to the guidelines for systematic review and meta-analysis of prediction models (11). This study protocol was registered on PROSPERO (registration number: CRD42023486813).

### 2.1 Search strategy

The databases searched included PubMed, Web of Science, Embase, CINAHL, MEDLINE, SinoMed, China National Knowledge Internet (CNKI) and Wan-Fang database, which were searched from the inception of the databases until 30 September 2025 in English and Chinese. Additional relevant studies were identified by reviewing the bibliographies of acquired studies and related review articles. Search terms were customized for each database to increase search precision. The Supplementary File details the specific search methodology employed, using PubMed as an illustrative example.

For the systematic review, the PICOTS framework was utilized, following recommendations from the CHARMS checklist for the Critical Appraisal and Data Extraction in Systematic Reviews of Prediction Modelling Studies. This framework aids in clarifying the review's aims, developing the search methods, and setting the parameters for what studies should be included or excluded. The essential elements of our systematic review are outlined subsequently.

P (Population): Preterm infants.

I (Intervention model): Any risk prediction model that incorporates multiple clinical variables to forecast the incidence of SIVH.

C (Comparator): Not applicable.

O (Outcome): Probability of developing SIVH during the hospital stay.

T (Timing): After evaluating the preterm infant's mother's antenatal clinical condition, prenatal treatment strategies, information during delivery, and information after the preterm infant is born, predict the outcome. Since nearly all cases of SIVH occur within one week after birth in premature infants, any information obtained within this timeframe can be included as variables in the model.

S (Setting): The intended use of the risk prediction model is to individualize the prediction of SIVH in preterm infants, enhancing the execution of preventative strategies to mitigate the occurrence of adverse events.

### 2.2 Inclusion and exclusion criteria

The inclusion criteria for the studies were as follows: (1) study subjects are preterm infants with a gestational age between 21 weeks and 37 weeks; (2) the primary outcome of interest is SIVH; (3) at least two risk prediction factors were used in constructing the model; (4) includes studies that conduct validation of a prediction model, whether through internal validation techniques (e.g., bootstrap, cross-validation, split-sample) or external validation (e.g., temporal, geographical), especially when such validation leads to model refinement or updates; (5) study types can include case-control or cohort studies. The lower bound of 21 weeks was selected as the inclusion criterion to maximize sensitivity and ensure that no potentially relevant studies were missed, particularly those involving infants at the margin of viability. This approach ensured a comprehensive search while maintaining clinical relevance in the final synthesis.

The exclusion criteria were: (1) studies that do not describe the process or methods of model construction, (2) studies that only analyze risk factors without establishing a predictive model, (3) duplicate publications or literature that is inaccessible for full-text retrieval. A systematic search for grey literature was not conducted. This decision was based on the assumption that exploring eight databases would be adequate to capture the majority of formally published, peer-reviewed prediction models for SIVH. Furthermore, systematic reviews of clinical prediction models often focus on published literature, as grey literature may contain studies with incomplete reporting, which could impede thorough data extraction and risk-of-bias assessment. This approach balances comprehensiveness with the feasibility of conducting a methodologically rigorous appraisal.

### 2.3 Study selection and screening

Study screening mainly includes three steps. Initially, duplicate studies were removed. Further screening was conducted on titles and abstracts to assess their eligibility. Subsequently, two independent reviewers conducted examination of the full text, screening the literature according to predetermined inclusion and exclusion criteria. Additionally, the reference lists of all qualifying studies were thoroughly examined to identify any potentially relevant studies. In cases of disagreement regarding the selection of studies, a discussion among three authors (FS, QQC, and ZHZ) was conducted to reach a consensus.

### 2.4 Data extraction

Data extraction will be carried out using a predefined form by two separate reviewers (FS, QQC), in line with CHARMS Checklist protocols. Any differences in reviewer opinions were settled through consensus. The essential elements for extraction encompass11 categories: data origin, participant details, predicted outcomes, potential predictors, cohort size, missing information, model creation, model performance, model evaluation, results, and interpretation and discussion. This comprehensive compilation of data will assist in assessing the bias risk and relevance of each study. Additionally, the collected general information about the studies includes the authors' names and publication years.

### 2.5 Assessment of risk of bias and applicability

The study's risk of bias and relevance are evaluated using the Prediction Model Risk of Bias Assessment Tool (PROBAST) (12). This evaluation was performed independently by two reviewers (FS and QQC). The PROBAST tool comprises 20 signaling questions across four domains: participants (evaluating bias and relevance issues associated with participant data and selection), predictor (examining biases and relevance in the definitions and measurements of predictors), outcome (assessing biases and relevance concerning outcome definitions and determinations), and analysis (reviewing biases and relevance issues in relation to statistical methods or analysis). Answer options include “yes,” “probably yes,” “probably no,” “no,” or “no information,” where “no” or “probably no” suggest a significant bias risk in that domain. The presence of significant bias in any area deems the study at a high bias risk overall. Relevance, excluding the analysis category, is judged on participants, predictors, and outcomes, with potential judgments being “low concern,” “high concern,” or “unclear concern” regarding relevance. Notably, only bias, not relevance, is evaluated within the analysis section. Any disagreements between the two reviewers were resolved through consensus discussion, with involvement of a third reviewer (ZHZ).

### 2.6 Data synthesis and statistical analysis

The synthesis of results from the included studies was conducted in two stages. First, a narrative synthesis was performed for all included models, summarizing their key characteristics. Second, we planned a quantitative meta-analysis using metrics such as the area under the curve (AUC), calibration slope, and Brier score to evaluate both discrimination and calibration. For specific measures where quantitative pooling was not feasible, findings were summarized narratively.

Perform meta-analysis of the AUC values for model validation using MedCalc software (version 15.0; MedCalc Software 2017, USA). Extract AUC values, standard errors, and 95% confidence interval (CI) for comprehensive analysis of results. If the study only reports the AUC and its 95% CI for the model, standard errors are calculated using the method proposed by Robert G (13). Test heterogeneity using the I^2^ index and Cochrane Q test (14), I^2^ is used to quantify the proportion of total variation that is attributable to heterogeneity (15). If *P* < 0.05 or I^2^ > 50%, consider the presence of heterogeneity and use a Random Effects Model (REM); if *P* ≥ 0.05 and I^2^ ≤ 50%, consider insignificant heterogeneity and use a Fixed Effects Model (FEM). Utilize the funnel plot and Egger's test to evaluate publication bias, where a *p*-value greater than 0.05 indicates a lower likelihood of publication bias (16). We are unable to perform a meta-analysis of the calibration due to the unavailability of relevant data. To explore potential sources of heterogeneity across the studies, we performed subgroup analyses focusing on study duration, external validation status, methods for predictor selection, and the final predictors incorporated into the models.

## 3 Results

### 3.1 Literature search

**Figure 1:**
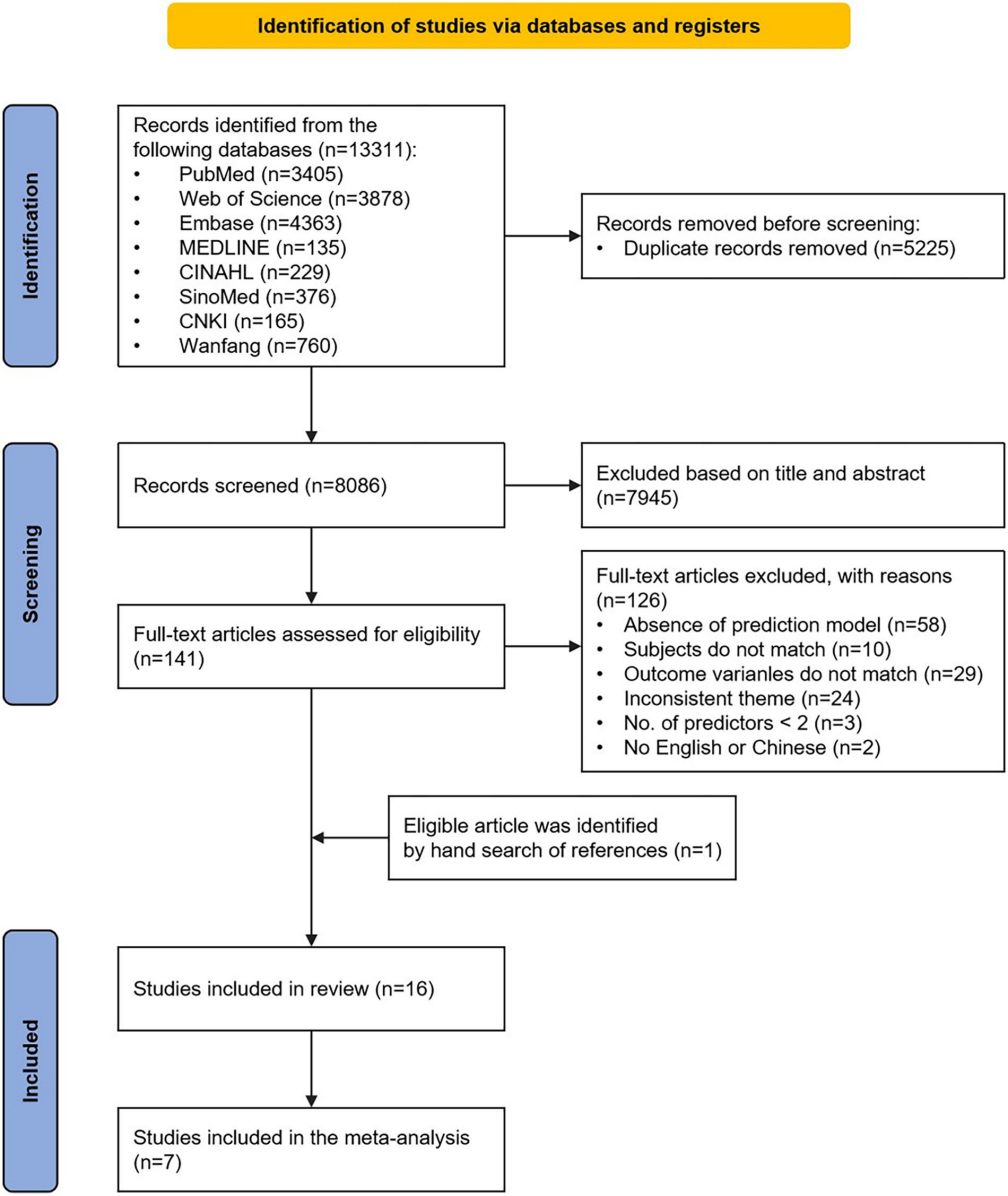
Flow diagram of the study selection process.

Figure 1 shows the comprehensive search process and results. The initial search resulted in 13,311 indexed records. Following the removal of 5,225 duplicate records identified across all databases, a total of 8,086 titles and abstracts underwent screening to assess their eligibility. Following this screening process, 141 studies were retained for full test assessment. We also found one study from references. During the subsequent evaluation, 16 studies with 16 models were included in this review. Due to inadequate reporting of model development details in the included studies, only 7 models ultimately met the inclusion criteria for the meta-analysis.

### 3.2 Study characteristics

Table 1 summarizes the study designs and participant characteristics of the 16 included studies (17–32). The publication timeline of these studies spans a considerable period, with the earliest dated 1998 and the most recent in 2025. 8 studies were published within the last five years. Most studies utilized data from multicenter neonatal research networks within their respective regions. With the exception of one study described as a retrospective case-control clinical study, all others employed a retrospective cohort design. The study periods of these cohorts varied widely, ranging from 2 to 11 years, with 10 cohorts having a study period exceeding 5 years. The study population predominantly consisted of preterm infants with a birth weight <1,500 g or a gestational age <32 weeks. In total, the 16 studies included 75,499 preterm infants from 14 regions. The sample sizes ranged from 328 to 30,973. The distribution of sample sizes was as follows: one study had <500 participants, four studies had 500–1,000, five studies had 1,001–5,000, four studies had 5,001–10,000, and two studies (sharing the same Japanese neonatal network) had >30,000 participants. The incidence of SIVH varied substantially across cohorts, ranging from 5.3% to 19.8%. Regarding model development, the events per variable (EPV) ratio ranged from 1.1 to 93.8. Nine studies had an EPV ≥20, while seven had an EPV <20. For the timing of predictor measurement, most studies assessed predictors within the first 3 days after birth, and only two studies developed models based solely on prenatal factors. The outcome (SIVH) was consistently defined according to the Papile or modified Papile criteria (1, 33), where mild IVH encompassed Grades I and II, and SIVH included Grades III and IV.

**Table 1:** Overview of basic data of the included severe IVH studies.

Author (year)	Country	Data source	Study design	Period of study	Participants	Main outcome	Time of outcome occurrence	Timing of predictor measurement	Severe IVH cases/sample size (%)	EPV
Zernikow B (17) (1998)	USA	2 NICUs in separate locations of the Vestische Kinderklinik-Witten/Herdecke University Hospital	Retrospective cohort study	April, 1990 to October, 1996	Preterm infants with birth weight < 1500 g or gestational age < 32 weeks	Severe IVH	Hospitalization period	-	79/865（9.13）	1.1
Heuchan AM (18) (2002)	Australian and New Zealand	24 neonatal medical centers in the Australian and New Zealand Neonatal Network	Retrospective cohort study	1995 to 1997	Preterm infants born at 24 to 30 weeks gestational age	Severe IVH	Hospitalization period	-	370/5413（6.84）	21.7
Linder N (19) (2003)	Israel	The neonatal department at the Rabin Medical Center	Retrospective case-control clinical study	January, 1995 to December, 1999	Preterm infants with birth weight < 1500g	Severe IVH	Hospitalization period	Within 24 h of birth	36/641 (5.62)	7.8
Vogtmann C (20) (2012)	Germany	23 hospitals in Saxony	Retrospective cohort study	2001 to 2005	Preterm infants with birth weight < 1500 g or gestational age < 32 weeks	Severe IVH	Hospitalization period	Within 72 h of birth	151/1782 (8.47)	5.0
Singh R (21) (2013)	USA	4 NICUs in Massachusetts	Retrospective cohort study	January, 2000 to December, 2010	Preterm infants born at 23 to 34 weeks gestational age	Severe IVH	Hospitalization period	Within 6 h of birth	163/2917 (5.59)	20.4
Luque MJ (22) (2014)	Argentina, Brazil, Chile, Paraguay, Peru and Uruguay	23 NICUs in the Neocosur Neonatal Network database of six South American countries	Retrospective cohort study	January, 2001 to December, 2011	Preterm infants with a birth weight of 500 to 1249g	Severe IVH	Hospitalization period	Within hours of birth	857/5747（14.91）	65.9
Lee J (23) (2018)	Korean	The multi-center database of the Korean Neonatal Network(without giving detailed Numbers)	Retrospective cohort study	January, 2013 to December, 2014	Preterm infants with birth weight < 1500g	Severe IVH	Hospitalization period	Within an hour of birth	239/2518（9.49）	23.9
He L (24) (2019)	China	3 large tertiary NICUs that belong to Guangzhou Women and Children's Medical Center	Retrospective cohort study	January 2015 to June 2018	Preterm infants born between 24 and 32 weeks GA with a BW from 600 to 1500 g	Severe IVH	Within 5 days after birth	Within 5 days after birth	41/615 (6.67)	1.3
Ushida T (25) (2021)	Japan	Approximately 200 neonatal departments within the Neonatal Research Network of Japan	Retrospective cohort study	January, 2006 to December, 2015	Preterm infants with gestational age of 22 to 32 weeks and birth weight ≤1500 g	Severe IVH	Hospitalization period	Antenatal	1642/30973（5.3）	93.8
Chawla S (26) (2022)	USA	18 NICUs in the Eunice Kennedy Shriver National Institute of Child Health and Human Development Neonatal Research Network	Retrospective cohort study	July, 2012 to August, 2017	Preterm infants born at 22 to 26 weeks gestational age	Severe IVH	28 days after birth	Within 12 h of birth	883/4455（19.82）	31.5
Ushida T (27) (2023)	Japan	Approximately 200 neonatal departments within the Neonatal Research Network of Japan	Retrospective cohort study	January, 2006 to December, 2015	Preterm infants with gestational age of 22 to 32 weeks and birth weight ≤1500 g	Severe IVH	Hospitalization period	Antenatal	1642/30973（5.3）	93.8
Kumar P (28) (2023)	USA	2 Level IV NICUs within the Vermont Oxford Network	Retrospective cohort study	January 1, 2011 to December 31, 2015	Infants with gestational age ≥ 23 weeks and birth weight≤1500g	Severe IVH	Hospitalization period	Within hours of birth	83/923 (8.99)	3.5
Yang YH (29) (2024)	China	33 neonatal medical centers in the Taiwan Neonatal Network	Retrospective cohort study	2016 to 2022	infants with birth weights ranging from 401 to 1500 g or gestational ages ranging from 22 weeks 0 days to 29 weeks 6 days	Severe IVH	Hospitalization period	Within 72 h of birth	591/7471 (7.91)	23.2
Kim HH (30) (2024)	Korean	77 neonatal medical centers in the Korean Neonatal Network registry database	Retrospective cohort study	2013 to 2020	Preterm infants with birth weight < 1500g	Severe IVH	Hospitalization period	Within 7 days after birth	826/9842（8.39）	37.6
Shen F (31) (2025)	China	The NICU in the Children's Hospital of Nanjing Medical University	Retrospective cohort study	June 2017 to December 2023	Preterm infants with birth weight < 1500g	Severe IVH	Within 7 days after birth	Within 24 h of birth	99/1009（9.81）	3.8
Hu Y (32) (2025)	China	The NICU of the pediatric unit of the First Affiliated Hospital of the Army Medical University in Chongqing	Retrospective cohort study	January 2017 to December 2023	Preterm infants born before 32 weeks’ gestation	Severe IVH	Within 7 days after birth	Within 48 h of birth	64/328（19.51）	2.2

“-”, not reported; IVH: intraventricular hemorrhage; EPV:Events Per Variable.

Table 2 summarizes the model information reported in the included studies. Based on the principle of selecting the best-performing model, only the single model identified as optimal within each of the 16 studies was included in our analysis. Regarding the handling of missing data, seven studies failed to report relevant information, eight studies reported directly deleting missing data prior to modeling, and only one study reported the absence of any missing data. Concerning the treatment of variable types, seven studies chose to dichotomize or categorize continuous predictors for model development. The methods for predictor selection varied considerably: ten studies employed univariable analysis for initial screening; two utilized LASSO regression; two applied machine learning-based techniques; one used an attribute selection radar chart based on information gain attribute evaluation; and one entered all candidate predictors directly into a stepwise logistic regression procedure. The number of final predictors incorporated into the models ranged from 4 to 13. The most frequently identified risk factors included gestational age (*n* = 14), birth weight (*n* = 9), sex (*n* = 9), antenatal corticosteroid therapy (*n* = 9), 1-minute Apgar score (*n* = 7), 5-minute Apgar score (*n* = 6), and mode of delivery (*n* = 5). In terms of the primary modeling technique, twelve studies relied exclusively on logistic regression, while the remaining four studies employed both logistic regression and machine learning methods for model construction. Assessment of model calibration was not reported in five studies. Among those that reported it, seven studies evaluated calibration solely using the Hosmer-Lemeshow test, and four studies presented calibration plots.

**Table 2:** Overview of the information of the included prediction models.

Author (year)	Missing data handling	Continuous variable processing method	Selection of predictors	Variable selection	Model development method	Calibration method	Validation method	Final predictors	Model performance	Model presentation
Zernikow B (17) (1998)	-	Categorical variables	Univariable analysis	Stepwise logistic regression	Artificial Neural Network Logistic Regression	-	Internal validation	Gestational age Sex Delivery method Apgar score at 1 min Apgar score at 5 min Birthweight White race Capillary PaCO_2_ on admission Capillary pH on admission Transfer after birth Artificially ventilated during transport Premature rupture of membranes Emergency delivery	A: 0.935 B: Not shown	-
Heuchan AM (18) (2002)	-	Categorical variables	Univariable analysis	Enter method	Logistic Regression	-	Temporal validation	Gestational age Sex Apgar score at 1 min Antenatal corticosteroid treatment Transfer after birth	A:0.77 (95%CI:0.73–0.82) B:0.76 (95%CI:0.73–0.79)	-
Linder N (19) (2003)	Direct exclusion	Continuous variable	Univariable analysis	Stepwise logistic regression	Logistic Regression	-	-	Antenatal corticosteroid treatment Early sepsis Fertility treatment (including IVF) Low PaCO_2_ during first 24 h	A: 0.82	-
Vogtmann C (20) (2012)	Direct exclusion	Categorical variables	Univariable analysis	Stepwise selection	Logistic Regression	Hosmer-Lemeshow	Internal validation	Gestational age Apgar score at 1 min Perinatal infection(within 3 days) Absence of pathological Doppler findings during pregnancy The use of tocolytic agents	A: 0.877 B: 0.861	-
Singh R (21) (2013)	-	Continuous variable	Univariable analysis	Multivariate logistic regression analysis	Logistic Regression	Hosmer-Lemeshow	External validation	Gestational age Sex Delivery method Apgar score at 5 min Birthweight Antenatal corticosteroid treatment Birth Location	A:0.86 (95%CI:0.82–0.91) B: 0.82 (95%CI:0.78–0.86)	Network online computing tool
Luque MJ (22) (2014)	-	Continuous variable	Univariable analysis	Forward stepwise	Logistic Regression	Hosmer-Lemeshow	Internal validation & temporal validation	Gestational age Sex Delivery method Apgar score at 1 min Birthweight Antenatal corticosteroid treatment Mechanical ventilation Respiratory distress syndrome	A: 0.787 B: 0.764	Network online computing tool
Lee J (23) (2018)	Direct exclusion	Categorical variables	Univariable analysis	Backward method	Logistic Regression	Hosmer-Lemeshow	Internal validation	Gestational age Sex Apgar score at 5 min Birthweight Antenatal corticosteroid treatment Multiple gestation Blood BD value within the first hour after birth	A:0.791 (95%CI:0.759–0.823） B: Not shown	A clinical scoring system
He L (24) (2019)	-	Categorical variables	Univariable analysis	Multivariate logistic regression analysis	Logistic Regression	Hosmer-Lemeshow	Temporal validation	Gestational age Apgar score at 1 min Birthweight Antenatal corticosteroid treatment Mechanical ventilation Hypotension	A: 0.830 B: 0.853	A clinical scoring system
Ushida T (25) (2021)	Direct exclusion	Continuous variable	Univariable analysis	Stepwise forward selection	Logistic Regression	Hosmer-Lemeshowand Calibration plots	Internal validation	Gestational age Sex Antenatal corticosteroid treatment Premature rupture of membranes Hypertensive disorders of pregnancy Chorionicity (monochorionic or dichorionic)	A:0.78 (95%CI:0.75–0.80) B: Not shown	Formula of risk score obtained by partial regression coefficient of each factor
Chawla S (26) (2022)	-	Continuous variable	Univariable analysis	Backward elimination method	Logistic Regression	Hosmer-Lemeshow	Internal validation	Gestational age Sex Delivery method Antenatal corticosteroid treatment Epinephrine in delivery room	A:0.69 (95%CI:0.66–0.72) B: 0.69 (95%CI:0.65–0.74)	Formula of risk score obtained by partial regression coefficient of each factor
Ushida T (27) (2023)	Direct exclusion	Continuous variable	Machine learning	SHapley Additive exPlanations	Logistic Regression, Ridge Regression, Fully Connected Neural Networks Support Vector Machine, Random Forest, Adaptive Boosting, Gradient Boosting Decision Tree	-	Internal validation	Gestational age Sex Delivery method Birthweight Antenatal corticosteroid treatment Premature rupture of membranes Hypertensive disorders of pregnancy Chorionicity (monochorionic or dichorionic) Maternal age Parity Gestational diabetes mellitus/diabetes mellitus Clinical chorioamnionitis	A: 0.774 B: Not shown	-
Kumar P (28) (2023)	No missing data	Categorical variables	Stepwise logistic regression	Stepwise logistic regression	Logistic Regression	Hosmer-Lemeshow	Internal validation	Sex Apgar score at 1 min Birthweight Birth Location	A: 0.84 B: 0.77	A clinical scoring system
Yang YH (29) (2024)	Direct exclusion	Categorical variables	Radar charts of attribute selection with the information gain attribute evaluator	Radar charts of attribute selection with the information gain attribute evaluator	K-Nearest Neighbor, Decision Tree, Random Forest, Neural Network, Logistic Regression, Gradient Boosting	Calibration plots and mean Brier scores	Internal validation & Temporal validation	Gestational age Apgar score at 5 min Birthweight Mechanical ventilation	A: 0.82 B: 0.84	Formula of risk score obtained by partial regression coefficient of each factor
Kim HH (30) (2024)	Direct exclusion	Continuous variable	Machine learning	Feature importance of ensemble algorithms	Logistic Regression with Ridge Regulation, Random Forest, eXtreme Gradient Boosting	-	Internal validation	Gestational age Apgar score at 1 min Apgar score at 5 min Birthweight Premature rupture of membranes Multiple gestation Hypertensive disorders of pregnancy Respiratory distress syndrome Maternal age Hypotension Level of neonatal resuscitation Pulmonic hemorrhage	A: Not shown B: 0.62 (Stage 1) 0.86 (Stage 2) 0.86 (Stage 3)	-
Shen F (31) (2025)	-	Continuous variable	LASSO regression mode	Multivariate logistic regression analysis	Logistic Regression	Hosmer-Lemeshowand Calibration plots	Internal validation	Gestational age Max FiO_2_ Hematokrit on admission Platelet count on admission	A:0.884 (95%CI:0.843–0.924) B:0.903 (95%CI:0.870–0.936)(by bootstrap) 0.859 (95%CI:0.793–0.925)	Nomogram
Hu Y (32) (2025)	Direct exclusion	Continuous variable	LASSO regression mode	Multivariate logistic regression analysis	Logistic Regression	Calibration plots	Internal validation	Gestational age Apgar score at 5 min Septic shock Pulmonic hemorrhage Hemoglobin count Platelet count	A:0.877 (95%CI:0.815–0.939) B:0.838 (95%CI:0.712–0.964)	Nomogram

“-”, not reported; A, development cohort; B, validation cohort; BD, base deficit; IVF, *in vitro* fertilization.

### 3.3 Models' validation

Among all the models, two studies conducted both internal and external validation, ten performed internal validation only, three carried out external validation only, and one study focused solely on model development without validation. Among the 11 studies that performed internal validation, the split-sample method was the most frequently employed approach, followed by cross-validation and bootstrap validation. Of the five studies that utilized external validation, four adopted temporal validation, while only one employed geographical validation.

### 3.4 Results of quality assessment

Table 3 summarizes the risk of bias and applicability of the included studies. Table 4 provides a detailed breakdown of the signaling questions within the domains. All studies exhibit a high risk of bias primarily due to issues in the participant and analysis domains.

**Table 3:** PROBAST results of the included studies.

Included studies	Bias risk assessment results				Applicability assessment results			Overall	
	study Subjects	Predictors	Results	Analysis	study Subjects	Predictors	Result	Bias risk	Applicability concern
Zernikow B (17) (1998)	High	Low	Unclear	High	Low	Unclear	Low	High	Unclear
Heuchan AM (18)(2002)	High	Low	Unclear	High	Low	Unclear	Low	High	Unclear
Linder N (19)(2003)	High	Low	Low	High	Low	Low	Low	High	Low
Vogtmann C (20) (2012)	High	Low	Low	High	Low	Low	Low	High	Low
Singh R (21)(2013)	High	High	Low	High	Low	Low	Low	High	Low
Luque MJ (22)(2014)	High	Low	Low	High	Low	Low	Low	High	Low
Lee J (23)(2018)	High	Low	Low	High	Low	Low	Low	High	Low
He L (24)(2019)	High	Low	Low	High	Low	Low	Low	High	Low
Ushida T (25)(2021)	High	Low	Low	High	Low	Low	Low	High	Low
Chawla S (26) (2022)	High	Low	Low	High	Low	Low	Low	High	Low
Ushida T (27)(2023)	High	Unclear	Low	High	Low	Low	Low	High	Low
Kumar P (28)(2023)	High	Low	Low	High	Low	Low	Low	High	Low
Yang YH (29)(2024)	High	Unclear	Low	High	Low	Low	Low	High	Low
Kim HH (30)(2024)	High	Unclear	Low	High	Low	Low	Low	High	Low
Shen F (31)(2025)	High	Low	Low	High	Low	Low	Low	High	Low
Hu Y (32)(2025)	High	Low	Low	High	Low	Low	Low	High	Low

**Table 4:** PROBAST signaling questions for model development and validation analyses in the included studies.

PROBAST domain and signaling questions	Development analysis (*n* = 16)		
	Yes/probably yes [*n* (%)]	No/probably no [*n* (%)]	No information [*n* (%)]
1. PARTICIPANTS			
1.1 Were appropriate data sources used, e.g., cohort, RCT, or nested case–control study data?	——	16 (100.00%)	——
1.2 Were all inclusions and exclusions of participants appropriate?	16 (100.00%)	——	——
2. PREDICTORS			
2.1. Were predictors defined and assessed in a similar way for all participants?	16 (100.00%)	——	——
2.2 Were predictor assessments made without knowledge of outcome data?	16 (100.00%)	——	——
2.3. Are all predictors available at the time the model is intended to be used?	12 (75.00%)	1 (6.25%)	3 (18.75%)
3. OUTCOMES			
3.1. Was the outcome determined appropriately?	16 (100.00%)	——	——
3.2. Was a prespecified or standard outcome definition used?	16 (100.00%)	——	——
3.3. Were predictors excluded from the outcome definition?	16 (100.00%)	——	——
3.4. Was the outcome defined and determined in a similar way for all participants?	16 (100.00%)	——	——
3.5. Was the outcome determined without knowledge of predictor information?	16 (100.00%)	——	——
3.6. Was the time interval between predictor assessment and outcome determination appropriate?	14 (87.50%)	——	2 (12.50%)
4. ANALYSIS			
4.1. Were there a reasonable number of participants with the outcome?	9 (56.25%)	7 (43.75%)	——
4.2. Were continuous and categorical predictors handled appropriately?	9 (56.25%)	7 (43.75%)	——
4.3. Were all enrolled participants included in the analysis?	10 (62.50%)	6 (37.50%)	——
4.4. Were participants with missing data handled appropriately?	1 (6.25%)	7 (43.75%)	8 (50.00%)
4.5. Was selection of predictors based on univariable analysis avoided?	6 (37.50%)	10 (62.50%)	——
4.6 Were complexities in the data accounted for appropriately?	15 (93.75%)	1 (6.25%)	——
4.7 Were relevant model performance measures evaluated appropriately?	5 (31.25%)	7 (43.75%)	4 (25.00%)
4.8 Were model overfitting, underfitting, and optimism in model performance accounted for?	8 (50.00%)	8 (50.00%)	——
4.9 Do predictors and their assigned weights in the final model correspond to the results from the reported multivariable analysis?	9 (56.25%)	——	7 (43.75%)

RCT, randomized controlled trial.

In the Participants domain, while the participant inclusion and exclusion criteria were deemed appropriate across all studies (Risk of Bias 1.2), the overall risk of bias in this domain was judged as high for all studies because the data were derived exclusively from non-nested case-control or retrospective cohort designs (Risk of Bias 1.1).

The Risk of Bias in the Predictors domain was low, attributable to the consistent definition and assessment of predictors across all studies. As most studies utilized prospectively collected databases, predictors were recorded without knowledge of the outcome data. Regarding the effectiveness of the included predictors in the models (Risk of Bias 2.3), the majority of studies (*n* = 12/16) met the requirements. A small number of studies (*n* = 3/16) failed to report relevant information, and the study by Singh et al. (21) included a predictor that was not statistically significant (male sex, *P* = 0.94).

In the Outcome domain, all studies applied a standard definition and classification for SIVH, which did not incorporate any predictors and was uniformly applied to all participants. The timing of predictor assessment was considered appropriate in most studies, as they were measured within 72 h post-birth to predict SIVH occurring within the first postnatal week or over the entire hospitalization. However, two studies did not report the precise timing of predictor measurement, which introduced a risk of bias in this domain.

The bias introduced in the Analysis domain was the primary source of overall risk in both model development and validation. The nine signaling questions substantially elevated the risk assessment in this domain. This risk primarily stemmed from the inappropriate handling of missing data (Signaling Question 4.4), inadequate assessment of model calibration (Signaling Question 4.7), and suboptimal methods for predictor selection (Signaling Question 4.5). Specifically, 43.75% of studies opted to directly delete missing values, while 50.00% failed to report their methods for handling missing data. For model calibration, 43.75% of studies relied solely on the Hosmer-Lemeshow goodness-of-fit test, and 25.00% did not report any calibration performance. Furthermore, 62.50% of studies selected predictors based solely on univariable analysis. Additionally, regarding sample size (Signaling Question 4.1), 43.75% of studies were deemed unreasonable as their Events Per Variable (EPV) was below 20. An equal proportion (43.75%) inappropriately categorized continuous variables during model development (Signaling Question 4.2). In the model construction process, 37.50% of studies were judged to have inappropriately excluded participants (Signaling Question 4.3). For model validation, although most models employed internal and/or external validation, 50.00% of those performing internal validation used only the split-sample method, which potentially introduces a risk of bias.

The applicability of this study was assessed in terms of participants, predictive factors, and outcome domains. In two studies, there was no reported information regarding the applicability of predictive factors, making it unclear when the original studies evaluated the predictive factors. Consequently, these two aspects were rated as having unclear applicability. Participants and outcome domains were both rated as having a low risk of applicability.

### 3.5 Meta-analysis of validation models included in the review

**Figure 2:**
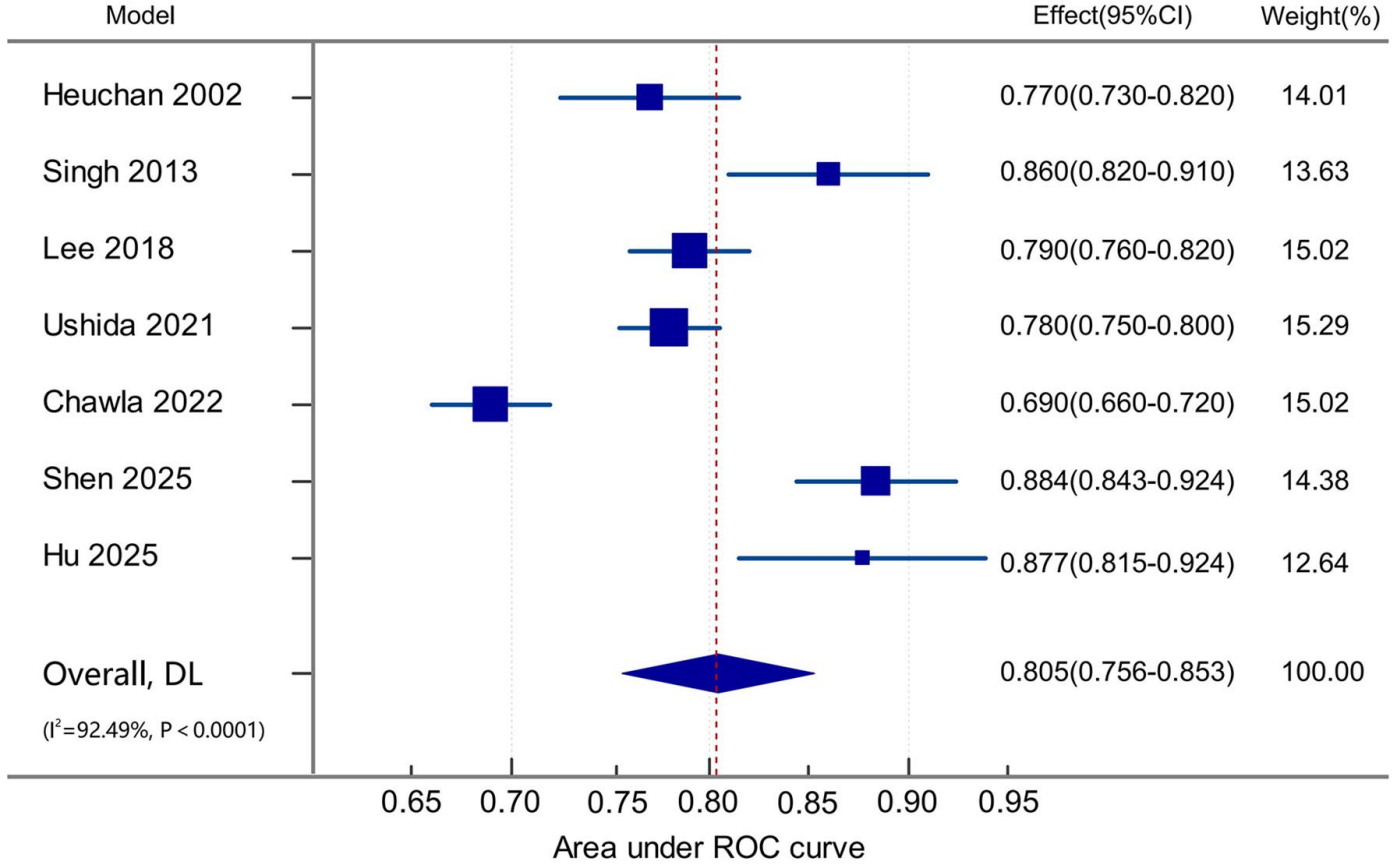
Forest plot of pooled area under the curve (AUC) for severe intraventricular hemorrhage prediction models.

**Figure 3:**
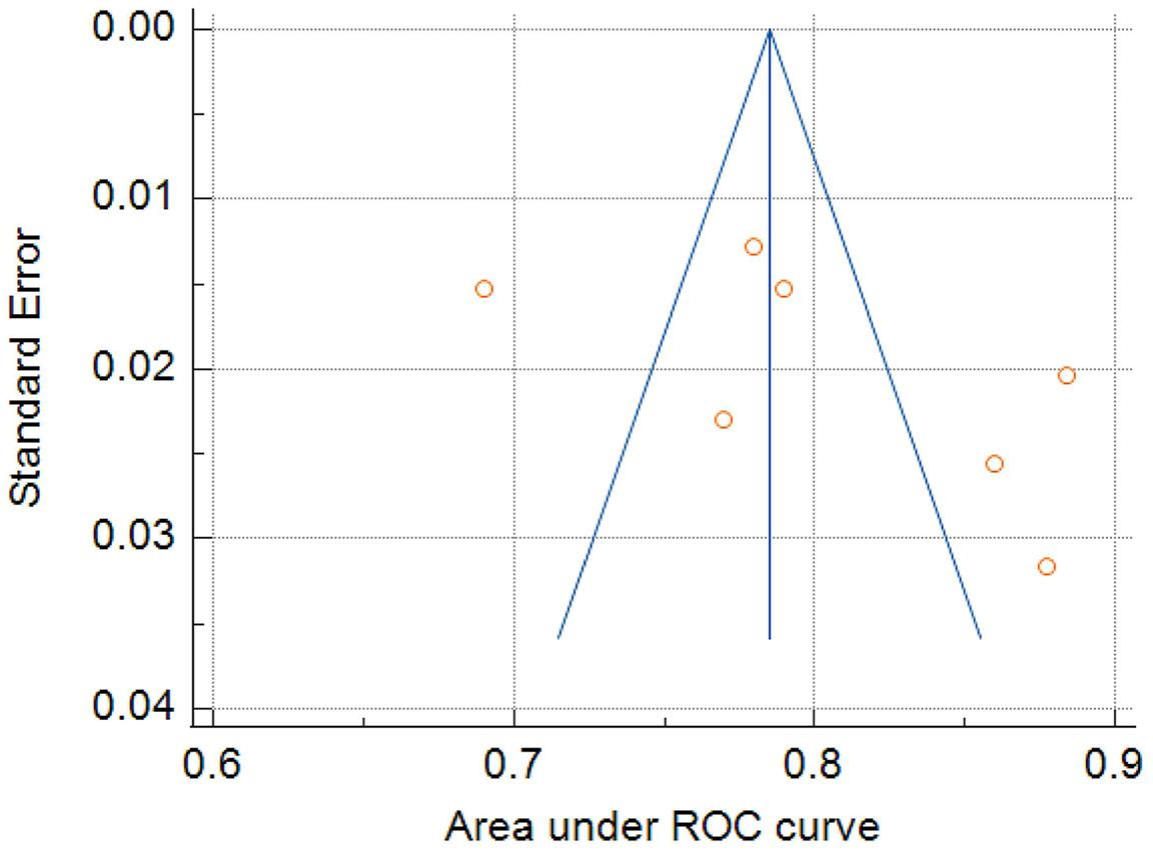
Funnel plot for publication bias assessment.

The AUC values for the 16 models included in this study ranged from 0.69 to 0.94, with only seven studies reporting the corresponding CI. Among these, one model had an AUC between 0.6 and 0.7, indicating poor discriminative ability; three models had an AUC between 0.7 and 0.8, indicating moderate discriminative ability; and three models had an AUC between 0.8 and 0.9, indicating good discriminative ability. The pooled AUC, calculated using a random-effects model, was 0.805 (95% CI: 0.756–0.853) (Figure 2). The heterogeneity index (I^2^) was 92.49% (*P* < 0.001), indicating substantial heterogeneity across the studies. Egger's test yielded a result of 6.7675 (*P* = 0.1876), suggesting no significant publication bias. The asymmetry observed in the funnel plot is likely attributable to the considerable heterogeneity among the studies rather than publication bias (Figure 3).

### 3.6 Subgroup analysis of 7 models included in the meta-analysis

Subgroup meta-analyses were performed to explore potential sources of heterogeneity. Pooled AUCs with 95% CI were calculated for each subgroup, and heterogeneity within subgroups was quantified using the I^2^ statistic. The I^2^ and *P* values indicated significant heterogeneity within most subgroups. Heterogeneity was absent only in subgroups characterized by a study duration of less than 5 years, the non-use of univariable analysis for predictor selection, and the absence of sex in the final predictor set. Models developed from consecutive samples demonstrated superior predictive performance compared to those that excluded certain study subjects (AUC: 0.828 vs. 0.774). Similarly, models derived from cohorts with a study period exceeding 5 years achieved higher AUC values than those from shorter-term cohorts (0.816 vs. 0.784). The method for predictor selection significantly influenced performance; models utilizing techniques like LASSO regression substantially outperformed those relying solely on univariable analysis (0.882 vs. 0.776). While externally validated models showed a higher predictive ability than non-validated models, the difference was marginal (0.814 vs. 0.801). The inclusion of specific predictors in the final model was associated with performance variations. Models incorporating sex as a predictor demonstrated lower performance than those without it (0.776 vs. 0.882). A similar negative association was observed for the inclusion of delivery mode (0.773 vs. 0.817). Conversely, the integration of hematological parameters into the final model was linked to significantly better predictive performance (0.846 vs. 0.773). These detailed results are presented in Table 5.

**Table 5:** Subgroup analysis of 7 models included in the meta-analysis.

Subgroup	Number of prediction models	AUC（95%*CI*)	*I^2^*(%)	*P*
Consecutive sample				
Yes (18, 23, 31, 32)	4	0.828 (0.778–0.877)	86.14	0.0001
No (21, 25, 26)	3	0.774 (0.697–0.851)	94.83	<0.0001
Research period				
>5 years (21, 25, 26, 31, 32)	5	0.816 (0.749–0.882)	94.96	<0.0001
≤5 years (18, 23)	2	0.784 (0.759–0.809)	0.00	0.4686
Univariate analysis method for screening predictive factors				
Yes (18, 21, 23, 25, 26)	5	0.776 (0.729–0.822)	90.46	<0.0001
No (31, 32)	2	0.882 (0.848–0.916)	0.00	0.8525
External validation				
Yes (18, 21)	2	0.814 (0.751–0.876)	85.46	0.0087
No (23, 25, 26, 31, 32)	5	0.801 (0.739–0.864)	94.32	<0.0001
The final predictive factor includes gender				
Yes (18, 21, 23, 25, 26)	5	0.776 (0.729–0.822)	90.46	<0.0001
No (31, 32)	2	0.882 (0.848–0.916)	0.00	0.8525
The final predictive factor includes the mode of delivery				
Yes (21, 26)	2	0.773 (0.655–0.890)	96.94	<0.0001
No (18, 23, 25, 31, 32)	5	0.817 (0.775–0.859)	85.33	<0.0001
The final predictive factors included hematological parameters				
Yes (23, 31, 32)	3	0.846 (0.794–0.898)	87.53	0.0003
No (18, 21, 25, 26)	4	0.773 (0.715–0.831)	92.29	<0.0001

AUC, area under the curve; CI, confidence intervals; SIVH, severe intraventricular hemorrhage.

## 4 Discussion

This study represents the first systematic review and assessment of all published prediction models for SIVH in preterm infants. The risk of bias assessment, conducted using the PROBAST tool, revealed that all included studies were rated as having a high risk of bias, which substantially limits the potential application of these models in current clinical practice. A meta-analysis of 7 validated models demonstrated a pooled AUC of 0.805 (95% CI: 0.756–0.853), indicating good discriminatory ability for predicting SIVH risk and suggesting potential clinical utility. However, significant heterogeneity was observed among the studies, necessitating further investigation into its sources. Future research should prioritize methodological improvements in several key areas, including the handling of missing data, the selection of predictors, and the calibration and validation of models. Furthermore, employing prospective study designs and adhering to the Transparent Reporting of a multivariable prediction model for Individual Prognosis Or Diagnosis, extended for artificial intelligence and machine learning applications reporting guideline is strongly recommended to enhance methodological rigor and minimize bias in future prediction model studies (34).

We evaluated 16 prediction models derived from 16 studies. With the exception of the study by Linder et al. (19), which employed a retrospective case-control design, all other studies utilized a retrospective cohort design. This introduced a high risk of bias in the Participants domain across all studies. The retrospective nature of these studies inherently limited the collection of potential predictor variables to those available within the existing databases, potentially necessitating the omission of other clinically important factors. Furthermore, although all models predicted the outcome of SIVH, the timing of SIVH measurement or diagnosis was unclear in some studies. Specifically, Singh et al. (21), Lee et al. (23), and Chawla et al. (26) explicitly stated in their reports that their retrospectively collected SIVH data did not distinguish between early-onset and late-onset cases. While the majority of IVH events occur in the early stage, other clinical or patient factors manifesting later could influence its occurrence. A minor proportion of late-onset cases could potentially affect model fitting. Future iterations of prediction models should consider these timing aspects and, where possible, adopt prospective study designs.

Another critical aspect warranting discussion pertains to the Analysis domain, which presented the highest risk of bias across all studies. Key sources of bias were identified in signaling questions 4.4, 4.5, 4.7, and 4.8. Specifically, 93.75% of models either directly deleted missing values or failed to report how missing data were handled—an approach only acceptable if data are missing completely at random and at a very low proportion. In most scenarios, this practice introduces selection bias, compromises sample representativeness, and leads to biased parameter estimates. Future studies should systematically report the patterns and proportion of missing data and consider advanced techniques such as multiple imputation to retain information and minimize bias (35). Inadequate or unreported assessment of model calibration was observed in 68.75% of the models. The widespread lack of proper calibration evaluation means we cannot be confident whether the predicted risks align with observed outcomes. A model with good discrimination but poor calibration may produce misleading predictions in clinical practice, ultimately compromising decision-making (36). Over-reliance on univariable analysis for predictor selection was another major concern, affecting 62.50% of the studies. This approach ignores complex multicollinearity among predictors, often retaining spuriously associated variables while omitting those that are meaningful only in multivariable contexts—ultimately undermining predictive performance. Future research should integrate both statistical and clinical relevance when selecting predictors. When numerous candidate variables are available, methods such as LASSO regression should be considered to eliminate non-informative variables, thereby avoiding oversimplification and bias introduced by univariable filtering (37). Regarding validation, half of the studies used only a simple split-sample approach for internal validation. This method is suboptimal as it often creates highly similar development and validation sets (38), tends to yield overoptimistic performance estimates, and its stability heavily depends on a single random partition. More robust techniques, such as bootstrap or k-fold cross-validation, should become standard practice. For instance, Vogtmann et al. (20) and Lee et al. (23) employed bootstrap validation, which has been shown to be an excellent approach for internal validation, particularly in small samples, helping ensure optimal efficiency in real-world applications (39).

Subgroup analysis served to elucidate sources of heterogeneity and yielded critical methodological and clinical insights for future prediction model development. Our key findings indicate that models based on consecutive samples, derived from longer-term cohorts, utilizing algorithms like LASSO regression for variable selection, and subjected to external validation consistently demonstrated superior predictive performance. Collectively, these findings underscore the fundamental impact of methodological rigor on model performance (40). At the level of data and modeling methodology, the superior performance of models based on consecutive samples confirms the importance of avoiding selection bias to ensure sample representativeness and model generalizability. Similarly, cohorts with longer follow-up periods captured outcome events more comprehensively, reducing misclassification due to inadequate follow-up and thereby yielding more robust model estimates. Most notably, models employing penalized algorithms such as LASSO regression for variable selection significantly outperformed those relying solely on univariable analysis. This suggests that moving beyond a univariable screening pre-selection step and directly adopting multivariable methods capable of handling multicollinearity and incorporating regularization may be a crucial pathway for enhancing both model performance and clinical validity (41). Regarding predictor selection, the subgroup analysis provided highly valuable clinical guidance. The observed performance decline associated with including sex and mode of delivery suggests these factors may have a weaker direct association with SIVH in preterm infants, or their predictive value might be superseded by other, more strongly associated covariates in the model. This finding advises researchers to base predictor selection on solid pathophysiological rationale and multivariable analysis results, rather than indiscriminately including all available variables. Conversely, the significant performance improvement seen with the inclusion of hematological parameters is highly instructive. It indicates that hematological markers reflecting neonatal coagulation status, circulatory state, or tissue oxygenation are likely deeply involved in the pathophysiology of SIVH. Future prediction model studies should prioritize the integration of such objective biomarkers with clear biological relevance. A finding warranting further discussion is that although externally validated models showed better performance, the magnitude of improvement over non-validated models was relatively modest. This might suggest a pervasive “optimistic bias” across the studies included in this systematic review, meaning that even the reported performance of non-externally validated models could be overestimated, potentially due to their internal validation methods (42). Therefore, establishing external validation as the gold standard and mandating its implementation remains an indispensable step in future research.

The existing predictive models reported in this review also have clinical significance. Models developed by Heuchan et al. (18) and Vogtmann et al. (20) are used as risk adjustment tools for neonatal medical centers in different countries, using IVH incidence as a quality indicator for neonatal care. This prompts management departments to conduct quality assessments after risk adjustment within the framework of external quality assurance for hospitals, facilitating improvements in the quality of neonatal care at both the hospital and national levels. Moreover, more than half of the included studies presented their prediction models in formats readily accessible to clinicians, such as clinical scoring systems, nomograms, or web-based calculators. This emphasis on practical presentation facilitates clinical application and decision-making at the bedside. Furthermore, most models require predictors that can be collected shortly after birth, and Ushida et al.'s model (25) can even predict before birth, as it does not include postnatal data. Indeed, such antenatal prediction holds significant potential for real-world healthcare applications, as it could provide a critical window for timely clinical interventions (43). After obtaining sufficient external validation, these models may be practically used to support clinical interpretation and decision-making, optimize perinatal care, and serve as an additional tool for parental consultation. Finally, high-frequency predictors have certain reference significance for nursing practice and future research. Some non-high-frequency predictors may also serve as potential predictors for future models, guiding research on future risk factors. Lee et al.'s study (23) was the first to use metabolic acidosis within 1 h after birth to assess the prediction of SIVH. After adding the pH value or base deficit (BD) value of arterial or capillary blood within 1 h after birth to the baseline model, the performance of the model was effectively improved. Some studies indirectly reflect that acidosis may be a useful predictor of IVH, as acidosis and hypoxia have been shown to directly damage brain vessels, leading to vascular rupture and bleeding (44). However, the results of blood gas analysis within the first hour after birth may also be influenced by other factors such as mechanical ventilation, and further research is needed to confirm this. Low gestational age and small birth weight have been proven to be independent risk factors for SIVH. The study by Linder et al. (19) was the first to closely match gestational age and birth weight between the SIVH group and the non-SIVH group. By reducing the confounding effects of gestational age and weight factors, the study detected an increase in the sensitivity of other variables affecting the incidence of IVH. Future research can build upon this foundation to further validate factors that are not yet clearly defined, potentially leading to medical interventions aimed at reducing the occurrence of SIVH.

## 5 Limitations

This study has certain limitations. Firstly, the combined AUC values are based on standard errors, and since most studies did not directly provide this metric, there is currently no other method to merge AUC values from existing indicators in the articles. Therefore, the combined AUC had to be calculated indirectly by inferring from standard errors, which may impact the accuracy of the results to some extent. Secondly, the AUC value should not be regarded as the sole metric for evaluating the predictive performance of the model; the model's calibration must also be taken into account. The models included in this study did not provide adequate data to support the meta-regression of calibration. As most of the included studies were conducted before corresponding guidelines were established to explicitly define the methods and steps for building predictive models (34), many studies lack information in the methodology section, which has somewhat affected the evaluation of the models. Future research should adhere to more rigorous methods and provide more transparent reporting. Additionally, a key methodological challenge inherent in most models included in this review is the potential for temporal overlap between predictor measurement and outcome occurrence. Although the included studies predominantly aimed to measure predictors shortly after birth, the exact timing of hemorrhagic onset is often difficult to ascertain from retrospective data. This ambiguity raises the possibility that certain predictors may have been measured after the hemorrhagic event had already begun, thereby representing a consequence rather than an antecedent risk factor. Such temporal ambiguity may result in models with good discriminative ability but limited etiological insight, and may lead to overestimation of predictive performance. We acknowledge this potential source of bias and emphasize that future prospective studies must carefully address this issue by clearly defining the chronological sequence between predictor assessment and outcome confirmation. Finally, although we attempted to identify the potential sources of heterogeneity in the study, the limited number of studies included in the analysis may introduce biases. Therefore, caution should be exercised when interpreting our findings.

## 6 Conclusion

This study represents the first comprehensive evaluation of the methodological quality and predictive performance of models for SIVH in preterm infants through systematic review and meta-analysis. The results demonstrate that existing models show good overall discriminative ability in distinguishing cases from non-cases (pooled AUC: 0.805), indicating their potential for clinical application. However, according to the PROBAST criteria, all included studies were judged to have a high risk of bias, primarily stemming from the inherent limitations of retrospective study designs and widespread methodological shortcomings in key analytical domains. Although current models provide a preliminary foundation for SIVH risk assessment, their present form is insufficient to directly and safely guide clinical decision-making. Future research must adhere to more rigorous methodological standards, focusing on optimizing approaches for handling missing data and selecting predictor variables, comprehensively reporting and evaluating model calibration, employing more robust validation strategies, and developing predictive models with low bias risk and clinical utility based on prospective study designs. Finally, actively pursuing external validation in independent cohorts remains an imperative final step to verify real-world performance and facilitate the translation of these models into practical clinical tools.

## Data Availability

Publicly available datasets were analyzed in this study. This data can be found here: The findings of this systematic review and meta-analysis are based on data extracted from previously published studies cited within the manuscript. Therefore, there is no single, original dataset or public repository link to be provided for this article.As detailed in the ‘Availability of data and materials’ section of the manuscript, the extracted data synthesized during this review (e.g., AUC values and their standard errors) are available from the corresponding author upon reasonable request.
